# Critical Care Utilization in Patients with Diabetic Ketoacidosis, Stroke, and Gastrointestinal Bleed: Two Hospitals Experience

**DOI:** 10.7759/cureus.4698

**Published:** 2019-05-21

**Authors:** Mohd Amer Alsamman, Samer Alsamman, Abdelmoniem Moustafa, Mohammad S Khan, Jenni Steinbrunner, Helen Koselka

**Affiliations:** 1 Hospital Medicine, The Warren Alpert Medical School of Brown University, Providence, USA; 2 Pulmonary / Critical Care, Ascension St. John Hospital, Detroit, USA; 3 Hospital Medicine, Miriam Hospital, Providence, USA; 4 Biostatistics, Good Samaritan Hospital, Cincinnati, USA; 5 Internal Medicine, Good Samaritan Hospital, Cincinnati, USA

**Keywords:** icu stay, health care cost, icu admission criteria

## Abstract

Introduction: Intensive Care Units (ICUs) are among the most expensive components of hospital care. Experts believe that ICUs are overused; however, hospitals vary in their ICU admission rate. Our hypothesis is based on clinical observations that many patients with diabetic ketoacidosis (DKA), stroke, and gastrointestinal (GI) bleeding admitted to the ICU don’t really need it and could be managed safely in a non-ICU level of care. Reducing inappropriate admissions would reduce healthcare costs and improve outcomes. Our primary objective was to determine the frequency of inappropriate ICU admissions. Secondary objectives were to evaluate which diagnoses were more unnecessarily admitted to the ICU, evaluate different variables and comorbidities, and determine the mortality rates during ICU admissions.

Methods: Patients admitted to the ICU, from the Emergency Department (ED) or transferred from the floor, during a one-year period were evaluated in this retrospective study. Patients 18-years old and above who had an admitting diagnosis of DKA, GI bleed, ischemic stroke, or hemorrhagic stroke were included. Patients in a comatose state, intubated, on vasopressors, hemodynamically unstable or had an unstable comorbid disease, subarachnoid hemorrhage, surgery during hospitalization prior to the ICU admission were excluded. Patients were categorized as having an appropriate or inappropriate ICU admission based on our institutional ICU admission criteria and data from available literature and guidelines.

Results: A total of 95 patients were included in our cohort. Seventy-two out of 95 (76%) were considered as inappropriate ICU admissions. When comparing each of the four admitting diagnoses, a significantly higher proportion of DKA patients were considered inappropriate ICU admissions when compared to the other diagnoses (*P* = 0.001). The overall mortality rate of ICU admissions was 16%, 15 patients out of 95 study population. When comparing each of the four admitting diagnoses, there was a significant difference in mortality rate with DKA having the lowest mortality (3%) and GI bleed having the highest mortality (43%). Out of the 15 patients who died, only 1 patient was categorized as an inappropriate ICU admission.

Conclusions: More than three-quarters of our study population was admitted to the ICU inappropriately. Incorporating severity scores in ICU admission criteria could improve the appropriateness of ICU admission and financial feasibility.

This article is based on a poster: Alsamman S, Alsamman MA, Castro M, Koselka H, Steinbrunner J: ICU admission patterns in patients with DKA, stroke and GI bleed: do they all need ICU? J Hosp Med. March 2015.

## Introduction

The intensive care unit (ICU) concept by today’s definition proliferated in the 1960s. Hospitals vary widely in their admission rates, which some experts believe are overused. Over the past 20 years, hospitals have reduced their number of non-critical-care beds overall by 35% while boosting the number of critical care units beds by a similar percentage [1]. The increased usage of ICU isn't always a positive development, as they are among the most expensive components of acute hospital care and, particularly among those who may not need it. It could increase patients' risk for complications and death [1]. Critical care use accounts for 1% of the US GDP, and 15% of all hospital costs [2-3].

Researchers examined trends in ICU admissions from hospital-based emergency departments (ED) using data from the National Hospital Ambulatory Care Survey from 2002 through 2009, and they found an increase from 2.79 million in 2002 and 2003 to 4.14 million in 2008 and 2009, a total increase of 48.8% and a mean biennial increase of 14.2%, while overall ED visits increased an average of 5.8% per biennial period [3].

In a retrospective cohort study from veterans affairs (VA) hospitals, the rate of ICU admissions ranged from 1.6% to 29.5%. About half of which had a 30-day predicted mortality of 2% or less [2].

A study in Critical Care Medicine evaluated every ketoacidosis (DKA) case in New York State and showed that 49% of those who are not admitted to ICU; neither had a greater length of stay (LOS) nor worse outcomes than those managed in ICU [4-5]. 

The Society of Critical Care Medicine has published guidelines for ICU admission and discharge, in an attempt to promote utilization of resources, through the use of models to formulate admission and triage criteria [6]. Each institution, in turn, should specify its policies by identifying the scope of services it provides [7].

We observed that certain categories of patients are frequently admitted to the critical care unit that may be equally served elsewhere with the majority being DKA, GI (gastrointestinal) bleed, and stroke. We assumed that unless there is a clear indication, many of those patients can be safely managed elsewhere.

Our primary objective was to determine the frequency of inappropriate critical care admissions. Secondary objectives were to evaluate which diagnoses were more unnecessarily admitted to the ICU, in relation to comorbid diagnosis and severity scores: Acute Physiology, Age, Chronic Health Evaluation II (APACHE II) [8] and Rockall [9]), and determine the mortality rates.

## Materials and methods

Using electronic medical records, we searched patients who were admitted to the critical care units at the two major hospitals in our system; in Cincinnati, Ohio from January 2014 to December 2014, both units are closed and managed by intensivists. One of the hospitals is a tertiary teaching hospital. The study population consisted of patients of 18-years or older with an admitting diagnosis of DKA, GI bleed, ischemic stroke, or hemorrhagic stroke. Patients with an unstable comorbid condition, subarachnoid hemorrhage, in a comatose state, intubated at admission, on vasopressors before ICU admission, post-tPA or post-surgery patients were excluded from the study. Institutional Review Board approval was obtained before conducting the study. This is a retrospective cohort study. 

Study variables included patient demographics, admitting diagnosis, comorbidities, APACHE II score, and Rockall score for GI bleed patients. For this study, admitting diagnosis was defined by the ICD-9 codes as follows: DKA - 250.1, 250.13; GI bleed - 578.0, 578.1, 578.9; stroke - 433, 434 435, 436, 437.

Our initial electronic medical record search showed that 155 patients with the diagnosis of DKA, GI bleed or stroke were admitted to the ICU. Patients were excluded for the following reasons: subarachnoid hemorrhage (19), intubated (19), comatose state (12), unstable comorbidity (8), use of vasopressors prior to ICU admission (1), and post-surgery (1). A total of 95 were eligible and included in the study.

Patients were categorized as an appropriate or inappropriate ICU admission based on the interventions done during ICU admission which were intubation, vasopressors, CPR, urgent surgical intervention related to primary diagnosis, ICP monitoring, and based on the following criteria that justified closer observation: respiratory rate ⩾40 or ⩽8 breaths/min, oxygen saturation <90% on ⩾50% oxygen, pulse rate <40 or >140 beats/min,systolic blood pressure <90 mm Hg, fall in level of consciousness (fall in Glasgow coma score >two points), repeated or prolonged seizures, pH less than 7.2 and rising arterial carbon dioxide tension with respiratory acidosis. Although there is no agreed upon universal ICU admission criteria, however, this categorization was based on available literature [6,10-11] combined with our institutional ICU admission criteria.

Basic descriptive analysis was done to describe the population and determine the frequency of appropriate and inappropriate ICU admissions based on our study definitions. Univariate analysis was conducted to evaluate the associations between predictors of complications in each diagnosis group (DKA, GI bleed, stroke) and an appropriate/inappropriate ICU admission. Chi-square test and Fisher’s exact test, when appropriate, were used for categorical variables and student t-tests were used for continuous variables. The significance level for all analysis was α = 0.05. Statistical analysis was performed using SPSS for Windows (SPSS Inc., Chicago, IL).

In order to assure quality in the chart review, a standardized data collection form was used. All results were reviewed at the end of the study by the primary investigator to avoid personal biases in data collection and minimize human errors.

## Results

**Figure 1:**
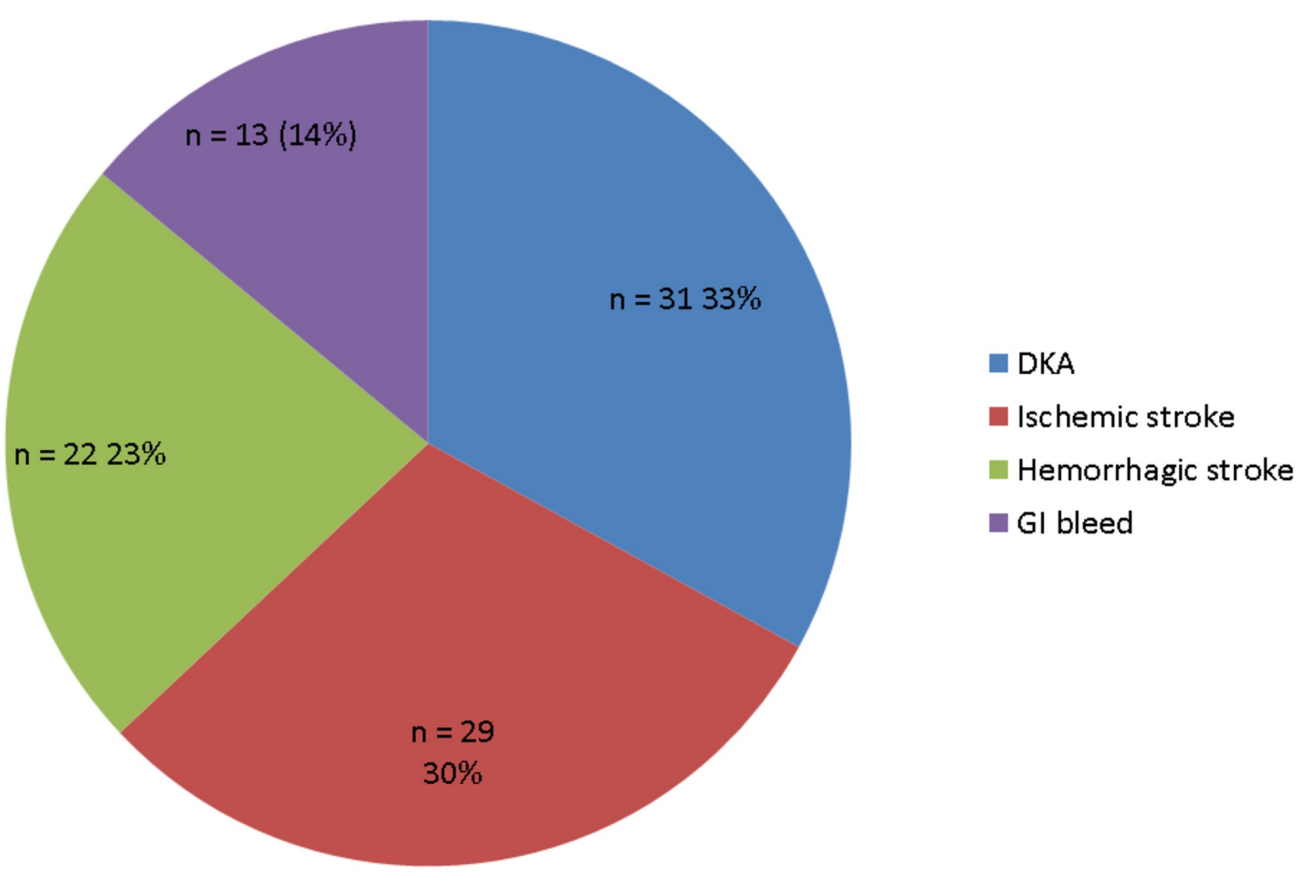
Percentage of admitting diagnosis to the intensive care unit DKA - diabetic ketoacidosis, GI bleed - gastrointestinal bleed

Among the 95 patients included in the study, 65% were male and 35% were female. The vast majority of patients (94%) were admitted to the ICU from the emergency department and 6% were transferred from another floor. Most patients had an admitting diagnosis of DKA (33%), followed by ischemic stroke (30%), hemorrhagic stroke (23%) and GI bleed (14%) (Figure 1).

Based on our study definitions, 23 cases (24%) were defined as appropriate and 72 cases (76%) were defined as inappropriate ICU admissions.

Univariate analysis showed a statistically significant association between having an admitting diagnosis of DKA and an inappropriate ICU admission (*P* < 0.001). Conversely, an admitting diagnosis of hemorrhagic stroke had a statistically significant association with appropriate ICU admission (*P* = 0.01) (Table 1). Additionally, we looked at different comorbidities and ICU admissions, and have found that patients with coronary artery disease (CAD) or chronic obstructive pulmonary disease (COPD) had higher rates of appropriate ICU admissions that were statistically significant (*P* = 0.02 and *P* = 0.03, respectively) (Table 2).

**Table 1:** Factors associated with appropriate and inappropriate intensive care unit admissions DKA - diabetic ketoacidosis, ED - emergency department, GI bleed - gastrointestinal bleed, CKD - chronic kidney disease, CAD - coronary artery disease, CHF - congestive heart failure, COPD - chronic obstructive pulmonary disease, Afib - atrial fibrillation

Factor	Appropriate N = 23		Inappropriate N = 72		P value
Admitting Diagnosis					
DKA, n (%)	0	0.0%	31	43.1%	<0.001*
GI bleed, n (%)	5	21.7%	9	12.5%	0.28
Ischemic stroke, n (%)	8	34.8%	21	29.2%	0.61
Hemorrhagic stroke, n (%)	10	43.5%	12	16.7%	0.008*
Admit Source *					0.35
Floor, n (%)	3	15.0%	4	6.1%	
ED, n (%)	17	85.0%	62	93.9%	
Gender					0.30
Male, n (%)	17	73.9%	44	62.0%	
Female, n (%)	6	26.1%	27	37.5%	
Comorbidities					
Diabetes, n (%)	9	39.1%	37	51.4%	0.31
CKD, n (%)	5	21.7%	6	8.3%	0.08
CAD, n (%)	7	30.4%	7	9.7%	0.02*
CHF, n (%)	5	21.7%	7	9.7%	0.13
COPD, n (%)	6	26.1%	6	8.3%	0.03*
Cancer, n (%)	4	17.4%	4	5.6%	0.08
Afib, n (%)	5	21.7%	9	12.5%	0.28
	Mean	St Dev	Mean	St Dev	P value
Age	68	7.5	55	15.4	0.002

**Table 2:** Comorbid conditions HTN - hypertension, DM - diabetes mellitus, CAD - coronary artery disease, Afib - atrial fibrillation, COPD - chronic obstructive pulmonary disease, CHF - congestive heart failure, CKD - chronic kidney disease

Comorbid Condition	N	%	P Value
HTN	64	67.4	0.2
DM	46	48.4	0.1
CAD	14	14.7	0.02 *
Afib	14	14.7	0.87
COPD	12	12.6	0.03 *
CHF	12	12.6	0.8
CKD	11	11.6	0.7
Cancer	8	8.4	0.5

When observing the mortality and survival rates of all cases, GI bleed had a significant association with mortality during hospitalization (*P* = 0.003). However, DKA had a significant association with survival during hospitalization (*P* = 0.02)., we found that only one DKA patient died during hospitalization, and it was due to other medical issues not related to DKA (Table 3). Both observations had a statistical significance. We did not observe any relationship between comorbidities and mortality rates. Six GI bleed patients died during hospitalization, three were considered to be appropriate ICU admissions and three were not. No statistical significance was observed between the two groups. 

**Table 3:** Mortality rates in relation to a different diagnosis DKA - diabetic ketoacidosis, GI bleed - gastrointestinal bleed

Factor	Died During Hospitalization N = 15		Survived During Hospitalization N = 80		P value
Admitting Diagnosis					
DKA, n (%)	1	6.7%	30	37.5%	0.02
GI bleed, n (%)	6	40.0%	8	10.0%	0.003
Stroke ischemic, n (%)	5	33.3%	23	28.8%	0.72
Stroke hemorrhagic, n (%)	3	20.0%	19	23.8%	0.75

Rockall score for GI bleed patients were examined, and only four patients had a score of 3 and all were considered to be appropriate (*P* = 0.01), yet there was no statistically significant relation to mortality (*P* = 0.98). Mean APACHE II score was 19.3 ± 8.8 (mean ± SD) for the whole study population, 17.7 ± 8.1 (mean ± SD) for the inappropriate group, and 26.1 ± 7.3 (mean ± SD) for the appropriate group, patients who were considered appropriate had significantly higher scores (*P* = 0.04) (Table 4).

**Table 4:** APACHE II score and correlation to the appropriateness of intensive care unit admission APACHE II - Acute Physiology, Age, Chronic Health Evaluation II

	Study population n = 95	Appropriate group n = 23	Inappropriate group n = 72	P Value
Mean ± SD	19.3 ± 8.8	26.1 ± 7.3	17.7 ± 8.1	0.04

We also studied the relationship between different variables such as low platelets in GI bleed, INR levels in both GI bleeds and hemorrhagic stroke and brain edema and their relation to appropriateness admission and mortality rates, however, we did not observe any statistical significance.

## Discussion

This study shows that 72% of the study group was inappropriately admitted to the ICU. There has been drastic growth in critical care beds at US hospitals [1]. This trend is partially due to hospital efforts to accommodate the escalating growth of the elderly population. However, a massive amount of ICU beds can lead to an upsurge of inappropriate usage of critical care services. This scenario will only decrease efficiency, increase hospital costs, and work against initiatives aimed to support healthcare reform [6].

The current study reports substantial overuse of ICU admissions at our hospitals, especially among patients with DKA. One important implication is that given the high cost of ICU admissions, having such a high number of patients inappropriately admitted necessitates the need to revise the ICU admission criteria at the hospitals. Part of the problem is the workload that falls on the nursing teams in the wards, which make it very difficult and unsafe for them to manage drips such as insulin drip that is used to treat DKA patients. Additionally, some stroke and GI bleed patients will require more frequent neurovascular assessments and more vital sign checks.

Having a step-down unit which could provide some level of intensive care could be a practical option that would reduce healthcare costs substantially, where intravenous drips could be managed.

We found that our data support the overall trend that there is a clear overuse of ICU resources and an increase in inappropriate admission for DKA patients and ischemic stroke patients, without any associated reduction in mortality. However, our study is first to incorporate severity score; APACHE and GI bleed score; Rockall, examine comorbid conditions, and find a relation between CAD, COPD, and likelihood of ICU need. Utilization of severity scores as part of admission criteria could potentially improve the appropriateness of ICU admission and financial feasibility.

The possible limitations of our study are the retrospective design; the lack of financial correlation that would incentivize organizations and hospitals to take more serious steps when it comes to savings; and limited sample size due to the recent implementation of the electronic medical record system.

## Conclusions

This study indicates that most DKA and hemorrhagic stroke patients are inappropriately admitted to the ICU. Those patients could be managed in a step-down or medical floors without affecting mortality. This would result in a substantial reduction in healthcare cost both for hospitals and patients. High APACHE II score, Rockall scores in GI bleeders, CAD, and COPD could predict the need for ICU admission.
